# Synthesis of a Novel α-Glucosyl Ginsenoside F1 by Cyclodextrin Glucanotransferase and Its In Vitro Cosmetic Applications

**DOI:** 10.3390/biom8040142

**Published:** 2018-11-10

**Authors:** Seong Soo Moon, Hye Jin Lee, Ramya Mathiyalagan, Yu Jin Kim, Dong Uk Yang, Dae Young Lee, Jin Woo Min, Zuly Jimenez, Deok Chun Yang

**Affiliations:** 1 Department of Oriental Medicinal Biotechnology, College of Life Science, Kyung Hee University, 1 Seocheon-dong, Giheung-gu, Yongin-si, Gyeonggi-do 17104, Korea; ssm8656@hanmail.net (S.S.M.); serendipity27@nate.com (H.J.L.); yujinkim@khu.ac.kr (Y.J.K.); rudckfeo23@naver.com (D.U.Y.); hero304@khu.ac.kr (J.W.M.); 2 Graduate School of Biotechnology, College of Life Science, Kyung Hee University, 1 Seocheon-dong, Giheung-gu, Yongin-si, Gyeonggi-do 17104, Korea; ramyabinfo@gmail.com (R.M.); zejp78@gmail.com (Z.J.); 3 K-gen (corp), 218, Gajeong-ro, Yuseong-gu, Daejeon 34129, Korea; 4 Department of Herbal Crop Research, National Institute of Horticultural and Herbal Science, RDA, Eumseong 27709, Korea; dylee0809@gmail.com

**Keywords:** cyclodextrin glycosyltransferase, cyclodextrin glycosyltransferase (CGTase), ginsenoside F1, α-glucosyl ginsenoside F1

## Abstract

Ginsenosides from *Panax ginseng* (Korean ginseng) are unique triterpenoidal saponins that are considered to be responsible for most of the pharmacological activities of *P. ginseng*. However, the various linkage positions cause different pharmacological activities. In this context, we aimed to synthesize new derivatives of ginsenosides with unusual linkages that show enhanced pharmacological activities. Novel α-glycosylated derivatives of ginsenoside F1 were synthesized from transglycosylation reactions of dextrin (sugar donor) and ginsenoside F1 (acceptor) by the successive actions of Toruzyme^®^3.0L, a cyclodextrin glucanotransferase. One of the resultant products was isolated and identified as (20*S*)-3β,6α,12β-trihydroxydammar-24ene-(20-*O*-β-*D*-glucopyranosyl-(1→2)-α-*D*-glucopyranoside) by various spectroscopic characterization techniques of fast atom bombardment-mass spectrometry (FAB-MS), infrared spectroscopy (IR), proton-nuclear magnetic resonance (^1^H-NMR), ^13^C-NMR, gradient heteronuclear single quantum coherence (gHSQC), and gradient heteronuclear multiple bond coherence (gHMBC). As expected, the novel α-glycosylated ginsenoside F1 (G1-F1) exhibited increased solubility, lower cytotoxicity toward human dermal fibroblast cells (HDF), and higher tyrosinase activity and ultraviolet A (UVA)-induced inhibitory activity against matrix metalloproteinase-1 (MMP-1) than ginsenoside F1. Since F1 has been reported as an antiaging and antioxidant agent, the enhanced efficacies of the novel α-glycosylated ginsenoside F1 suggest that it might be useful in cosmetic applications after screening.

## 1. Introduction

Ginseng saponins, referred to as ginsenosides, are one of the major bioactive substances of *Panax ginseng* Meyer, a commonly used traditional herbal medicine in Korea, China, and Japan. Ginsenosides have been reported to have antifatigue and antioxidant activities, improve brain function, enhance stamina, and regulate blood circulation with approval from the Korea Food and Drug Administration (KFDA), in addition to various other pharmacological activities including anticancer [1,2], anti-inflammation [3], and antidiabetes [1,4] functions. These various pharmacological activities of ginsenosides typically depend on the types of sugar moieties and the position and linkage of their attachment [4,5]. More than 289 distinct saponins had been identified from different *Panax* sp. up to 2012 [6], and these compounds show different biological activities based on structural differences [3].

Ginsenosides are mainly classified as protopanaxadiol-type (PPD), protopanaxatriol-type (PPT), and oleanane-type saponins and further grouped into major and minor saponins based on the position and linkages of sugar moieties. The minor saponins, which are ginsenoside metabolites, are responsible for most of the pharmacological activities of ginseng which include ginsenoside F1, Rh1, compound K, and Rh2 [7]. These ginsenosides are mainly absorbed into systemic circulation [8]. Ginsenoside F1 is a minor saponin from the leaf of *P. ginseng* that was reported to have skin whitening activity [9], modulate skin diseases [10], and function as an antiaging and antioxidant agent [11], suggesting that it might be a candidate for cosmetic applications.

Synthesis of novel and diversified compounds is a way to extend the efficacy of natural products. Such diversity can be generated by biosynthetic reactions such as glucosylation [12,13]. Especially, enzymatic glycosylation provides more regioselectiveness than conventional chemical synthesis [14]. A number of reports have suggested that transglycosylation by enzymes can be used to improve physiochemical functions such as taste, solubility in water, and oxidative stability of numerous active substances [15,16]. Among these enzymes, cyclodextrin glycosyltransferase (CGTase, 1,4-α-*D*-glucan: 1,4-α-*D*-glycopyranosyltransferase, cyclizing, EC 2.4.1.19) [17] has been reported to accelerate reactions between natural products and starch hydrolysate or β-cyclodextrin to produce glucosylated modifications of natural compounds such as hesperidin, glycosylglycerol [14,16] rutin [18], and steroidal saponins [19].

Although the beta isomer was prominent, the alpha isomer has attracted much attention in recent years. The increased solubility of hesperidin [16,18] and decreased bitterness of glycosylated stevioside [15] by CGTase was reported. Other studies reported the mild sweet taste with no odor, no tongue-pricking, and increased stability of *O*-α-glucosylthiamin compared with thiamin hydrochloride [20] and the powerful skin whitening activity of alpha arbutin [21] compared with beta arbutin [22] as a result of glycosylation by CGTase.

In this study, we aimed to synthesize the unusual alpha glycosylated ginsenoside F1 by a reaction involving ginsenoside F1, dextrin, and CGTase. One of the resultant novel compounds was purified, and the structure was elucidated by various nuclear magnetic resonance (NMR) spectra and Fourier-transform infrared spectroscopy (FTIR). We also evaluated the cytotoxicity and protective effect of α-glycosylated ginsenoside F1 against ultraviolet (UV) damage by measuring matrix metalloproteinase-1 (MMP-1) expression in human dermal fibroblast cells. In addition, the in vitro antityrosinase activity of α-glycosylated ginsenoside F1 was evaluated against mushroom tyrosinase.

## 2. Materials and Methods

### 2.1. Materials

Ginsenosides compound K (CK), Rh2, Rh1, F1, aglycone PPD (aPPD), and aglycone PPT (aPPT) were obtained from the laboratory of Hanbangbio, Kyung Hee University, South Korea. Toruzyme 3.0L (the crude enzyme of CGTase) obtained from Novozymes, China, was extracted from *Thermoanaerobacter* sp. Dextrin was supplied by Fluka Chemie AG (Buchs, Switzerland), and all the other chemicals used were of analytical grade and from commercial sources.

### 2.2. Biotransformation

The preliminary screening of glycosylation was carried out as the method of Wang et al., 2010 [19]. Different ginsenosides, CK (1.6 mM, 1 eq), Rh2 (1.6 mM, 1 eq), Rh1 (1.56 mM,1 eq), F1 (1.56 mM, 1 eq), aPPD (2.17 mM), and aPPT (2.09 mM) together with the sugar donor dextrin (9.9mM, 6 eq, 10–15 units of glucose) were dissolved in 20 mM sodium phosphate buffer (1 mL, pH 7.0). Next, 25 μL of Toruzyme^®^ 3.0L with initial activity of 3.0 KNU (kilo novo units)/g [17] was added to the reaction mixture and reacted at 50 °C for 2 h. and kept in boiling water for 5 min to inactivate the enzyme. The mixture was extracted three times with an equal volume of *n*-butanol, and the *n*-butanol layer was washed twice with distilled water to remove excess dextrin, dried in a rotary evaporator under vacuum [19], and dissolved in methanol for thin-layer chromatography (TLC).

### 2.3. Glycosylation of Ginsenoside F1

For further experimental analysis, F1 was used as a substrate. The effects of different concentrations of dextrin (0–7 mg) and Toruzyme^®^ (5–30 μL) and different reaction durations (0.5–3 h) on specificity of F1 glycosylation were examined using the procedure described above. For purification of glycosylated F1, F1 (500 mg, 1.56 mM, 1 eq) and dextrin (2g, 7.92 mM, 5 eq) were dissolved in 500 mL of 20 mM sodium phosphate buffer and then treated with 15 mL of Toruzyme^®^ 3.0 L.

### 2.4. Identification of Glycosylated Ginsenoside F1

Semiqualitative screening of the glycosylated products was carried out by TLC and high-performance liquid chromatography (HPLC) was carried out by Ramya et al., 2015 and Quan et al., 2012 [13,23] with slight modifications. TLC was performed with silica gel plates (60 F254, Merck, Darmstadt, Germany) using the developing solvent CHCl_3_:CH_3_OH:H_2_O (65:35:10, *v*/*v*, lower phase). The TLC plates were dried, dipped in 10% H_2_SO_4_, and air dried with heating at 110 to 120 °C. The HPLC analysis was carried out on an Agilent 1260 series with a C_18_ (250 × 4.6 mm, ID 5 µm) column using distilled water as solvent A and acetonitrile as solvent B mobile phases. The following gradient was used: A:B ratios of 80.5:19.5 for 0–29 min, 70:30 for 29–36 min, 68:32 for 36–45 min, 66:34 for 45–47 min, 64.5:35.5 for 47–49 min, 0:100 for 49–61 min, and 80.5:19.5 for 61–66 min with a flow rate of 1.6 mL/min. The sample was detected at a wavelength of 203 nm.

### 2.5. Nuclear Magnetic Resonance Analysis

Structural elucidation of the new compound by NMR spectra (^1^H NMR, ^13^C NMR, gHSQC (heteronuclear single quantum correlation) and heteronuclear multiple bond correlation (gHMBC)) were performed using a Varian Unity INOVA AS 400 FT-NMR spectrometer (Varian, Palo Alto, CA, USA), and chemical shifts were expressed in δ (ppm), with tetramethylsilane (TMS) used as an internal standard. The dimethyl sulfoxide-d_6_ (DMSO-d_6_) was used as a solvent. Melting points were obtained using a Fisher-John’s melting point apparatus. Optical rotations were measured on a JASCO P-1010 digital polarimeter. Infrared spectra were obtained on a Perkin Elmer Spectrum One FTIR spectrometer (Perkin-Elmer, Walthanm, MA, USA). High resolution fast-atom bombardment mass spectrometry (HR-FAB/MS) were recorded using a JEOL JMS-700 (JEOL, Tokyo, Japan) mass spectrometer.

### 2.6. Cell Lines and Cell Culture

Human dermal fibroblasts (HDF) were purchased from the Korean Cell Line Bank (Seoul, Korea). The cells were grown in Dulbecco’s modified essential media (DMEM) supplemented with 10% fetal bovine serum (FBS) and 1% penicillin–streptomycin at 37 °C in a humidified atmosphere containing 95% air and 5% CO_2_.

#### 2.6.1. Ultraviolet Irradiation and Sample Treatment

A high-pressure metal halide lamp (UVASUN 3000, Mutzhas, Munich, Germany) emitting wavelengths in the range of 340 to 450 nm was used as a UV source. Human dermal fibroblasts cells were seeded at 4 × 10 cell/dish in 60-mm culture dishes for 24 h. Prior to UV irradiation, cells were washed twice with phosphate buffer saline (PBS), and the medium was replaced with 1 mL of PBS. The incident dose at the surface of the cells was 66 mW/s. The spectral distribution of the UVASUN 3000 source was determined with a Beckman UV 5270 spectrophotometer (Beckman, Munich, Germany, FRG).

#### 2.6.2. Cytotoxicity Assay

Human dermal fibroblasts cells were cultured at a density of 1 × 10^4^ cells/well in 96-well flat-bottomed plates in a 5% CO_2_ humidified atmosphere at 37 °C. After 24 h of culture, the medium was exchanged with medium containing different concentrations of ginsenoside F1 (F1) and α-glycosylated ginsenoside F1 (Glycosylated F1), and the cells were incubated for a further 24 h. Cell viability was determined by the 3-(4,5-dimethylthiazol-2-yl)-2,5-diphenyltetrazolium bromide (MTT) assay [24] with slight modification. Briefly, 10 μL of MTT solution (5 mg/mL) was added to each well and incubated for 4 h. After removal of MTT, the cells were lysed with 100 μL DMSO, and absorbance was measured at 570 nm using a microplate reader (Bio-Tek Instruments, Winooski, VT, USA).

#### 2.6.3. In Vitro Tyrosinase Inhibition Activity

Tyrosinase from *Agricus bisporus* (mushroom) was purchased from Sigma Chemicals Co. (St Louis, MO, USA). Inhibition of tyrosinase activity was measured as previously described [22]. L-DOPA (3-(3,4-dihydroxyphenyl)-L-alanine, 0.83 or 3.3 mM) was used as the substrate, and 600 units of tyrosinase was added in the presence or absence of F1, glycosylated F1, or arbutin. The absorbance was measured at 475 nm in a microplate reader (Bio-Tek Instruments, Winooski, VT, USA).

#### 2.6.4. Assay for Inhibition of Matrix Metalloproteinase-1 Expression

Matrix metalloproteinase-1 (MMP-1) level was quantified using a sandwich ELISA Quantikine total human MMP-1 kit (R&D Systems Inc., Minneapolis, MN, USA) After UV irradiation, HDF cells were cultured in DMEM with F1, and glycosylated F1, or ((−)-*cis*-3,3′,4′,5,5′,7-hexahydroxy-flavane-3-gallate) (EGCG) as a positive control. The culture supernatants were harvested, and MMP-1 was measured according to the manufacturer’s instructions. Absorbance was measured at 490 nm in a microplate reader microplate reader (Bio-Tek Instruments, Winooski, VT, USA).

## 3. Results and Discussion

### 3.1. Biotransformation of Minor Ginsenosides by Cyclodextrin glycosyltransferase (CGTase)

Among the major ginsenosides, Rb1, Rc, Re, and Rg1 have already been used as substrates for the synthesis of series of new α-glycosylginsenosides through transglycosylation [13,25,26]. However, after oral administration, the major ginsenosides were converted into minor ginsenosides by intestinal microflora. Therefore, we used minor ginsenosides CK, Rh2, F1, Rh1, aPPD, and aPPT as acceptors with dextrin as a sugar donor during CGTase enzyme transglycosylation. As a result, CK, Rh2, F1, and Rh1 yielded new transglycosylated compounds with different retention factor (R_f_) values compared with known ginsenoside standards (Figure S1). Among these, PPT type ginsenosides Rh1 and F1 showed more glycosylated products, possibly due to the glucose attached to α-OH at C-6 and another –OH at C-20 of the dammerendiol steroidal aglycone. We chose F1 for further studies because of the distinct separation of glycosylated products in addition to its previous reported application in cosmetics and skin care. PPD and PPT aglycone did not generate glycosylated products, indicating that sugar molecules are primarily involved in transglycosylation.

### 3.2. Specificity of Transglycosylation of Ginsenoside F1

Even though the effects of various factors on transglycosylation by Toruzyme were already reported [19,27], this should be validated for the effective synthesis of new compounds. Therefore, the effects of different concentrations of dextrin and CGTase (Toruzyme) on the degree of glycosylation were investigated by HPLC. As shown in Figure S2a, the 5:1 *w*/*w* ratio of dextrin: F1 showed the highest yield. There was no significant difference for greater than five volumes, and it was difficult to separate saponin after biotransformation due to the combined extraction of sugar with saponin in the recovery process. In addition, increasing the amount of enzyme rapidly increased the yield up to 20 μL of enzyme with 1 mg of F1 and 5 mg of dextrin, as determined by HPLC (Figure S2b).

### 3.3. Transglycosylation Analysis of Ginsenoside F1

**Figure 1:**
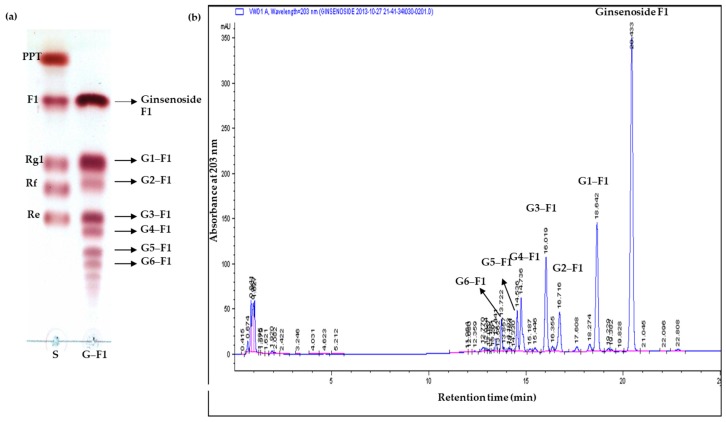
(**a**) Thin layer chromatography of new F1 glycosylated products after removal of excess sugar. (**b**) High-performance liquid chromatography (HPLC) analysis of F1 and various glycosylated products after reaction. G1–F1, compound **1**; G2–F1, compound **2**; G3–F1, compound **3**; G4–F1, compound **4**; G5–F1, compound **5**; G6–F1, compound **6**.

The glycosylation of F1 with dextrin and CGTase for different time durations yielded several new spots that appeared below F1 on TLC (Figure S3). The reaction products were washed several times with water to remove the unreacted excess sugar molecules. The six new spots (G1–F1, G2–F1, G3–F1, G4–F1, G5–F1, and G6–F1) under ginsenoside F1 on TLC (Figure 1a) and the corresponding peaks (G1–F1, G2–F1, G3–F1, G4–F1, G5–F1, and G6–F1), other than ginsenoside F1 on HPLC analysis (Figure 1b), were considered new glycosylated products from F1. G1–F1 (R_f_ = 0.53) on TLC was isolated as a pure form by silica gel chromatography and elution with CHCl_3_/CH_3_OH (9:1). The yield of compound G1–F1 was 12% (74 mg) and the structure was identified by ^1^H-NMR, ^13^C-NMR, and two-dimensional (2D) NMR and by correlations with the HSQC and HMBC spectra. The low percentage of yield is due to the formation of other products (G2–F1, G3F1, G4–F1, G5–F1, and G6–F1).

**Figure 2:**
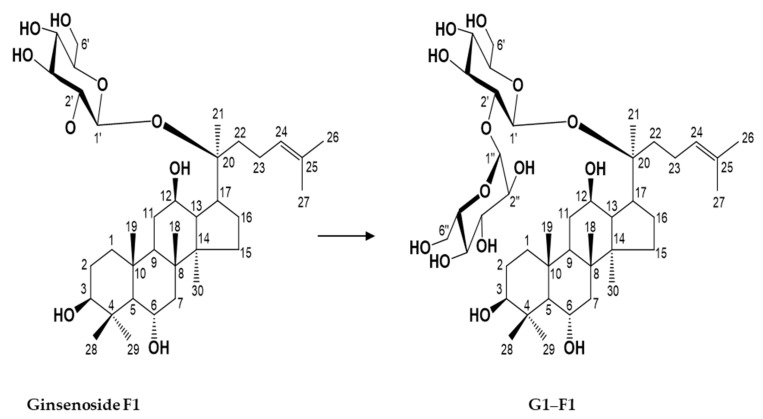
Chemical structures of ginsenoside F1 and its α-glycosylated F1 (G1–F1).

Compound **1** (G1–F1) was obtained as a white powder. The molecular formula of G1–F1 was determined to be C_42_H_72_O_14_ from the pseudomolecule ion peak *m*/*z* 799.4843 [M-H]^-^ in negative high-resolution fast atom bombardment-mass spectrometry (FAB-MS). The infrared spectrum showed strong absorbance from hydroxyl groups (3366 cm^–1^) and a double bond (1650 cm^–1^) in G1–F1 (Figure S4). In the ^1^H NMR spectrum, proton signals of one olefin methine (δ_H_ 5.30, dd, *J* = 6.0, 6.4 Hz, H-24), three oxygenated methines (δ_H_ 3.48, H-3; 4.10, H-12; 4.38, H-6), and eight singlet methyls (δ_H_ 1.98 (H-28), 1.58 (H-26), 1.56 (H-27), 1.55 (H-21), 1.45 (H-29), 1.08 (H-18), 1.01 (H-19), 0.98 (H-30)) were observed, indicating that G1–F1 has a protopanaxatriol-type triterpene moiety. Proton signals due to the sugar moiety, two anomeric proton signals at δ_H_ 5.81 (d, *J* = 3.6 Hz, H-1′′) and 5.04 (d, *J* = 8.0 Hz, H-1′), and several oxygenated methines and methylene proton signals at δ_H_ 3.72~4.56 were observed (Figure S5a). The ^13^C NMR spectrum of G1–F1 (Figure S5b) exhibited 42 carbon signals due to a triterpene with two hexoses. An olefin quaternary carbon signal at δ_C_ 131.0 (C-25), one olefin methine carbon signal at δ_C_ 125.9 (C-24), one oxygenated quaternary carbon signal at 83.5 (C-20), three oxygenated methine carbon signals (δ_C_ 78.6 (C-3), 67.8 (C-6), 70.2 (C-12)), and eight methyl carbon signals (δ_C_ 32.0 (C-28), 25.7 (C-26), 22.3 (C-21), 17.8 (C-18), 17.6 (C-19), 17.5 (C-27, 30), 16.3 (C-29)) were observed for the protopanaxatriol-type aglycone moiety. The chemical shifts of the sugar moieties signal (δ_C_ 98.1 (C-1′), 81.2 (C-2′), 78.5 (C-3′), 76.6 (C-5′), 75.5 (C-4′), 62.1 (C-6′)) suggested the presence of a glucopyranoside. The coupling constant of the anomeric proton signal (δ_H_ 5.04, H-1′) was 8.0 Hz, confirming β-*D*-glucopyranoside. Another sugar moiety (δ_C_ 103.0 (C-1′′), 75.2 (C-3′′), 74.6 (C-2′′), 74.4 (C-5′′), 71.9 (C-4′′), 62.8 (C-6′′) suggested the presence of glucopyranoside; the coupling constant of the anomeric proton signal (δ_H_ 5.81, H-1′′) was 3.6 Hz, confirming that the glucopyranose had a α-glucosidic linkage. The connection between the β-*D*-glucopyranosyl unit (C-1′) and the C-20 of the aglycone and that of another α-*D*-glucopyranosyl unit (C-1′′) with C-2′ of the inner glucose was verified by the cross-peaks observed between the anomer proton signal at δ_H_ 5.04 (H-1′) and the oxygenated quaternary carbon signal at δ_C_ 83.5 (C-20) and between the anomer proton signal at δ_H_ 5.81 (H-1′′) and the oxygenated methine carbon signal at δ_C_ 81.2 (C-2′) in the HMBC spectrum, respectively (Figure S5c,d). This was confirmed by the downfield shifts of the carbon (δ_C_ 78.5 (C-3′)) and proton signals (δ_H_ 4.53 (H-3′)) due to the glycosylation effect. Ultimately, the structure of G1-F1 was determined to be (20S)-3β,6α,12β -trihydroxydammar-24-ene-(20-*O*-β-*D*-glucopyranosyl-(1→2)-α-*D*-glucopyranoside), which has not been reported previously (Figure 2).

### 3.4. Characterization of Novel α-Glycosylated Ginsenoside F1

#### Water Solubility of Ginsenoside F1 and Novel α-Glycosylated Ginsenoside F1

Transglycosylation reactions catalyzed by CGTase are an efficient method to enhance the water solubility of various compounds [16,18,28]. Accordingly, the water solubility of α-glycosylated ginsenoside F1 was higher than that of F1 alone (data not shown). The soluble α-glycosylated ginsenoside F1 should not only facilitate investigation of the pharmacological activities of ginsenoside F1, but also may be useful as a cosmetics ingredient.

### 3.5. Cell Cytotoxicity

#### 3.5.1. Comparison of Cell Viability of Ginsenoside F1 and Novel α-Glycosylated Ginsenoside F1 in Human Dermal Fibroblast Cells

**Figure 3:**
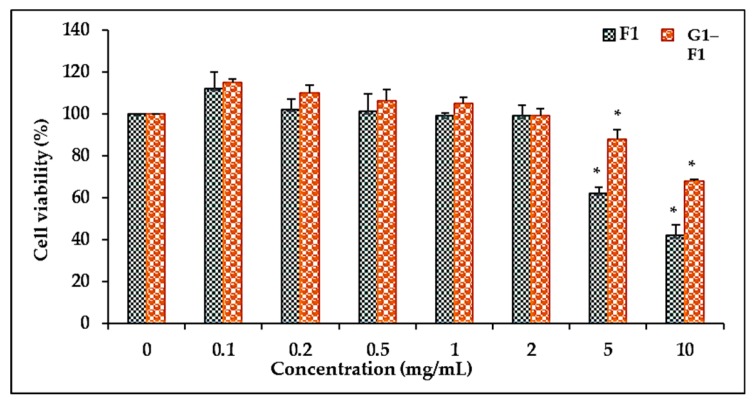
Cytotoxicity of ginsenoside F1 and α-glycosylated ginsenoside F1 in human dermal fibroblast cells. Cells were preincubated with or without compounds for 24 h, and cell viability was evaluated by 3-(4,5-dimethylthiazol-2-yl)-2,5-diphenyltetrazolium bromide (MTT) assay. Data represent the mean ± SD (standard deviation) of triplicate experiments. * *p* < 0.05 compared with the control. F1: ginsenoside F1; G1–F1: α-glycosylated ginsenoside F1.

To evaluate the effects of α-glycosylated ginsenoside F1 and ginsenoside F1 on the cell viability of HDFs, the cells were treated with different concentrations. Ginsenoside F1 reduced the cell viability of HDFs to a greater extent than α-glycosylated ginsenoside F1 (G1–F1) in a dose-dependent manner (Figure 3). The α-glycosylated ginsenoside F1 showed lower toxicity toward HDFs than ginsenoside F1 up to a concentration of 5 mg/mL. The cell viability was greater than 90% of that of the control cells up to 2 mg/mL. These results showed that ginsenoside F1 and α-glycosylated ginsenoside F1 have no significant cytotoxicity against skin cells. Thus, the inhibitory effect of these compounds on collagenase expression was not due to cytotoxicity of these compounds at concentrations up to 2 mg/mL.

#### 3.5.2. Inhibition of Tyrosinase Activity by Ginsenoside F1 and G1–F1

**Figure 4:**
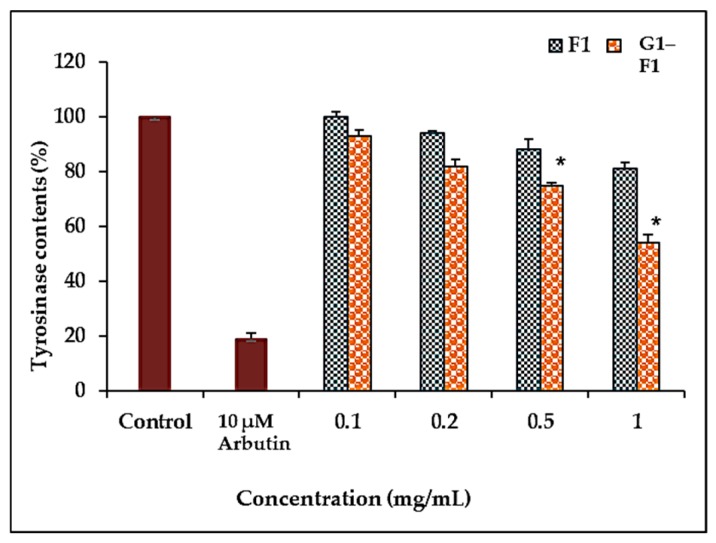
Inhibitory effects of ginsenoside F1 and α-glycosylated ginsenoside F1 on Mushroon tyrosinase activity. Tyrosinase activity was measured using 3.3 mM L-DOPA as a substrate. Results are expressed as the percentage of inhibition by ginsenoside F1 and α-glycosylated compound. Arbutin was used as a positive control. Data represent the mean ± SD of triplicate experiments. * *p* < 0.05 compared with the control. F1: ginsenoside F1; G1–F1: α-glycosylated ginsenoside F1.

To investigate the tyrosinase inhibitory activity of G1–F1, the half maximal inhibitory concentration (IC50) values against mushroom tyrosinase were measured. The tyrosinase inhibitory activity of α-glycosylated ginsenoside F1 was higher than that of ginsenoside F1 but weaker than that of arbutin (Figure 4).

It was previously reported that F1 can function as an anti-aging and antioxidant agent [11] and as a drug against skin cancer with antiproliferation and whitening functions [10]. Comparison of the inhibition of tyrosinase activity showed that α-glycosylated ginsenoside F1 had a greater inhibitory effect on tyrosinase activity than ginsenoside F1, indicating that α-glycosylated ginsenoside F1 might be an efficacious anti-tyrosinase agent for use in cosmetics.

#### 3.5.3. Inhibition of Ultraviolet A (UVA)-Induced Matrix Metalloproteinase- (MMP-1) Expression of Ginsenoside F1 and G1–F1

**Figure 5:**
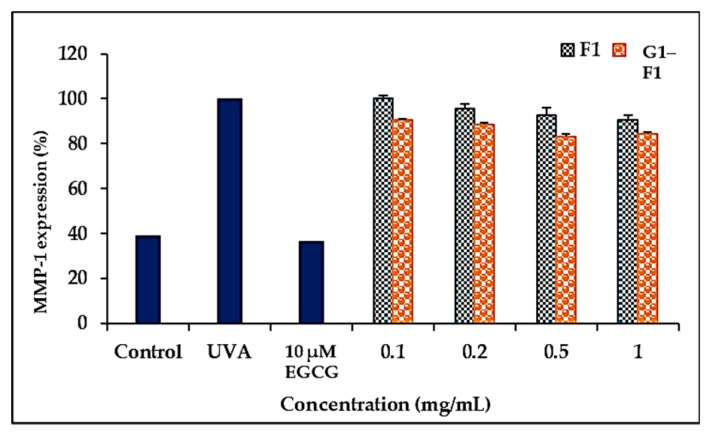
Inhibitory effects of ginsenoside F1 and α-glycosylated ginsenoside F1 on the expression of MMP-1 in UVA-irradiated human dermal fibroblasts. The cells were cultured in the presence of ginsenoside F1 and α-glycosylated ginsenoside F1 (0–1 mg/mL) for 24 h and subjected to ELISA. The results were expressed as the average ± SD of triplicate determinations. * *p* < 0.05 compared with UVA irradiation. F1: ginsenoside F1; G1–F1: α-glycosylated ginsenoside F1.

Skin aging occurs as a result of collagen degradation through induction of MMPs by UV irradiation [29]. The α-glycosylated ginsenoside F1 exhibited a greater inhibitory effect against collagenase (MMP-1) than the ginsenoside F1 after UVA irradiation of HDF cell lines (Figure 5), indicating that the C-3-hydroxyl group in the compounds is important for inhibitory activity. (−)-*cis*-3,3′,4′,5,5′,7-Hexahydroxy-flavane-3-gallate (EGCG) was used as a positive control.

In addition to the number of sugars, their linkage positions and alpha vs. beta linkages affect pharmacological activities. For example, ginsenoside F1 and Rh1 have the same number of sugar moieties and the same molecular weight but different glucose attachment positions at C-20 and C-6, respectively. F1 showed significantly greater inhibition of viability than Rh1 in prostate cancer cell lines [30]. The glycosylation and nano formulations of ginseng saponins [13,25,26,31,32,33] and other steroidal saponins [19,27] has recently attracted increased interest.

The alpha isomers of glucose also exhibited significant activity, especially stronger inhibitory activity of α-arbutin on tyrosinase compared with β-arbutin [22]. Similarly, in comparison with the common beta isomers of glucose in ginsenosides, α-glycosyl ginsenoside was reported to have a reduced bitter taste [26], suggesting its potential as an additive in food products.

## 4. Conclusions

This study describes for the first time the glycosylation of ginsenoside F1 by CGTase and identification of a novel α-glucosylated F1 with an unusual α-*D*-glcp-(1→2)-β-*D*-glcp sugar chain (G1–F1). The novel compound G1–F1 showed lower cytotoxicity and stronger inhibitory activity against tyrosinase and collagenase (MMP-1) than ginsenoside F1. This novel G1–F1 may be a potential pharmacological active compound. A single α-glucosylated F1 was purified in this study, and other new glycosylated spots remain to be characterized.

## Supplementary Materials

The following are available online at http://www.mdpi.com/2218-273X/8/4/142/s1. Figure S1: TLC analysis of transglycosylation of minor ginsenosides (Rh2, CK, PPD, Rh1, F1, PPT). S, standard; (i) Each ginsenoside with CGTase only and (ii) Each ginsenoside with CGTase and dextrin, Figure S2. (a). Effect of dextrin on transglycosylation of ginsenoside F1. Toruzyme^®^ (20 μL) was incubated with ginsenoside F1 (1 mg) in 1 mL of 20 mM sodium phosphate buffer at 60 ± 1°C for 2 h, (b). Effect of Toruzyme^®^ on transglycosylation of ginsenoside F1. Toruzyme^®^ (5–30 μL) was incubated with ginsenoside F1 (1 mg) and dextrin (5 mg) in 1 mL of 20 mM sodium phosphate buffer at 60 ± 1°C for 2 h. The yield was calculated as the increase in product by HPLC, Figure S3: TLC analysis of transglycosylation of ginsenoside F1 with Toruzyme^®^ and dextrin at different times (min). S, standard of higher molecular weight ginsenosides than F1 in the same protopanaxatriol type ginsenosides; F1+toru (Control 1): Ginsenoside F1 and CGTase Toruzyme enzyme alone; Dextrin+toru (Control 2): Dextrin (sugar donor) and CGTase Toruzyme enzyme alone, Figure S4. (a). Infrared spectrum (FTIR) of novel α-glycosylated ginsenoside F1 (G1–F1), Figure S5. (a). ^1^H-NMR spectrum of novel α-glycosylated ginsenoside F1 (G1–F1). (b). ^13^C-NMR spectrum of novel α-glycosylated ginsenoside F1 (G1–F1). (c). gHSQC spectrum of novel α-glycosylated ginsenoside F1 (G1–F1). (d). Key gHMBC spectrum of novel α-glycosylated ginsenoside F1 (G1–F1).
